# Acute myocardial infarction occurring while on chronic clopidogrel therapy (‘clopidogrel failure’) is associated with high incidence of clopidogrel poor responsiveness and stent thrombosis

**DOI:** 10.1371/journal.pone.0195504

**Published:** 2018-04-06

**Authors:** Ehud Regev, Elad Asher, Paul Fefer, Roy Beigel, Israel Mazin, Shlomi Matetzky, For The Platelets and Thrombosis in Sheba (PLATIS) - Study Group

**Affiliations:** Leviev Heart Center, Sheba Medical Center, Tel Hashomer, Sackler Faculty of Medicine, Tel Aviv University, Tel Aviv, Israel; University of Bologna, ITALY

## Abstract

**Objectives:**

The clinical significance of the laboratory-based phenomenon of clopidogrel hypo-responsiveness and platelet reactivity associated with acute myocardial infarction, despite chronic clopidogrel therapy, is largely unknown. We aimed to determine platelet reactivity and clinical and angiographic features in 29 consecutive patients sustaining an acute myocardial infarction despite chronic (≥1 month) clopidogrel therapy.

**Methods:**

Platelet reactivity was determined on admission using conventional aggregometry. All patients underwent coronary angiography within 24 hours of admission. Patients were matched with clopidogrel-naïve acute myocardial infarction patients. Clopidogrel-naïve patients received a 600 mg clopidogrel loading dose and 75 mg/day thereafter.

**Results:**

Of the 29 study patients, 19 (66%) presented with ST-elevation myocardial infarction, and in 25% the infarction was related to angiographically-proved definite stent thrombosis. Two-thirds of these patients were poor responders to clopidogrel (adenosine diphosphate-induced platelet aggregation >50%) and dual antiplatelet poor responsiveness was found in 57% in the chronic clopidogrel therapy group. Compared with clopidogrel-naïve patients, chronic clopidogrel therapy patients were more likely to demonstrate clopidogrel poor responsiveness (66% versus 38%, p = 0.02), to be diabetic (52% versus 33%, p = 0.1) and to have multi-vessel coronary disease (79% versus 55%, p = 0.03).

**Conclusions:**

Patients sustaining acute coronary syndrome despite chronic clopidogrel therapy are more likely to exhibit inadequate platelet inhibition with clopidogrel.

## Introduction

Antiplatelet therapy is central in the treatment of patients with acute coronary syndrome (ACS) and in patients undergoing percutaneous coronary intervention (PCI). Accordingly, dual antiplatelet therapy with aspirin and P2Y12 blockers has become the standard care in these patients [1]. However, despite the use of dual antiplatelet therapy, adverse cardiovascular events continue to occur [2]. The thienopyridine derivative clopidogrel inhibits platelets by blocking adenosine diphosphate (ADP)-mediated platelet activation, and has been shown by prospective large-scale studies to reduce the risk of recurrent cardiovascular events [3–5]. Nevertheless, platelet hypo-responsiveness to clopidogrel has been described in up to 25% of patients treated with clopidogrel, and is associated with high rates of recurrent ischemic and thrombotic events [6–13]. However, while clopidogrel hypo-responsiveness is a well-studied laboratory-based pharmacodynamic phenomenon, there is only a limited amount of data regarding the clinically defined phenomenon ‘clopidogrel failure’, which is the occurrence of an ischemic event during clopidogrel therapy [6]. Although clopidogrel is widely used [14], the clinical significance of ‘clopidogrel failure’, as well as platelet responsiveness associated with this phenomenon, are largely unknown. The aim of the current study was to describe the clinical and angiographic characteristics, as well as the platelet reactivity of patients sustaining ACS while on chronic clopidogrel therapy.

## Materials and methods

### Study design

The study comprised 29 patients on chronic (i.e. >1 month) clopidogrel therapy who presented with an acute myocardial infarction (MI) within 12 hours of symptom onset. All patients were treated with clopidogrel 75 mg/day and aspirin 100mg/day prior to the index event. Diagnosis of acute MI was based on troponin I elevation and typical anginal pain and/or electrocardiographic changes suggestive of acute ischemia. All patients were treated with aspirin 300mg and atorvastatin 80mg on admission. All patients with ST-segment elevation MI (STEMI) underwent immediate angiography and primary PCI. Patients with non-STEMI (NSTEMI) underwent coronary angiography and PCI within 24 hours of admission.

Patient baseline characteristics as well as their prior medical therapy were recorded on admission. Use of clopidogrel and compliance were recorded based on patients’ reports on admission, and also by reviewing patients’ prescribed medications from their HMO medical records. Demographic, historical, clinical and angiographic data of all patients, as well as prior medical therapies, were recorded. Coronary angiography findings, including the number of diseased coronary vessels and location of the culprit lesion, were determined by two blinded interventional cardiologists. Stent thrombosis was diagnosed based on the Academic Research Consortium criteria for probable or definite stent thrombosis [15]. Culprit lesions in sites not previously treated with PCI were defined as de-novo stenosis.

### Blood extraction

Blood for platelet reactivity was drawn on admission with a loose tourniquet through a short venous catheter. These blood tests are taken routinely in all patients admitted with ACS in our institution. Blood was collected into tubes containing 3.2% sodium citrate and was assessed for platelet function immediately after it was drawn. Blood samples were centrifuged and the upper fraction collected as platelet-rich plasma. Remaining blood was centrifuged again to obtain platelet poor plasma.

### Platelet function assessment

Platelet aggregation was evaluated by a turbidimetric PACKS-4 aggregometer (Helena Laboratories, Beaumont, TX, USA) using ADP (10 μM) and archidonic acid (1.6 mol/L) as agonists. Changes in light transmission were recorded for 5 minutes and maximal amplitude of aggregation was measured. All blood samples were evaluated in the same laboratory and by the same operator who was blinded to patient therapy and clinical status. In accord with previous studies [16], optimal response to clopidogrel was defined as ADP-induced Agg (max) less than 50%, and ADP-induced Agg (max) more than or equal to 50% as poor responsiveness. Aspirin non-responsiveness was defined by archidonic acid-induced platelet aggregation more than 20%, as previously suggested [17].

### Matching

Patients were matched on a ratio of 1:2 with consecutive clopidogrel-naïve patients, who were admitted with a diagnosis of acute MI and treated with a standard dose of clopidogrel (600/75mg/day) in addition to aspirin 300mg and atorvastatin 80 mg following their admission. The matched patients were derived from a large group of consecutive acute MI patients in whom platelet reactivity had been determined as part of the routine protocol. The chronic clopidogrel treatment group and the clopidogrel-naïve group were recruited concurrently. Matching was done for age, gender, type of acute MI (STEMI versus NSTEMI) and history of cardiovascular disease (past history of ACS\PCI or a cerebrovascular event). A separate matching was done for age, gender, type of acute MI, history of cardiovascular disease and diabetes mellitus. These patients were tested for platelet reactivity 48–72 hours post admission using the same methods as those outlined above.

### Data analysis

Continuous variables are presented as means ± standard deviation and t-test was used for comparison of the variables. Categorical variables were presented as percentages and were compared by Chi square or Fisher exact test as indicated. P less than 0.05 was considered statistically significant. Matching between the ‘clopidogrel failure’ patients and clopidogrel-naïve patients was performed by SAS software (version 8.2, SAS Institute Inc., Cary, NC, USA). Patients were matched on a 1:2 ratio for age, gender, type of acute MI and history of cardiovascular disease.

The study was reviewed and approved by the Institutional Review Board/Ethics Committee (IRB) of Sheba Medical Center at Tel Hashomer, Israel. All data were analyzed anonymously; therefore, the IRB waived the need for informed consent.

## Results

### Baseline characteristics

Of the 29 patients enrolled, 21 (72%) were male with a mean age of 70 ± 12 years. Fifteen (52%) suffered from diabetes mellitus. Patient baseline characteristics are presented in Table 1. Nineteen (66%) patients presented with acute STEMI and 10 (34%) with NSTEMI.

**Table 1:** Baseline characteristics of clopidogrel-failure and clopidogrel-naïve patients.

Baseline Characteristics	Clopidogrel- failure patients (n = 29)	Clopidogrel-naïve patients (n = 58)	P-value
Age	69±10	68±13	0.6
Gender (male)	21(72%)	41(72%)	1.0
Prior CAD^a^	22(76%)	44(76%)	0.5
Dyslipidemia	24(83%)	46(79%)	0.8
Smoking	6(21%)	16(27%)	0.6
Diabetes mellitus	15(52%)	19(33%)	0.1
Hypertension	26(89%)	47(81%)	0.4
STEMI^b^	19(66%)	38(67%)	1.0

^a^CAD = Coronary artery disease;

^b^STEMI = ST-elevation myocardial infarction

Indications for chronic clopidogrel treatment prior to the qualifying event were ischemic heart disease and a previous cerebrovascular accident/transient ischemic attack in 22 (76%) and 7 (24%) patients, respectively. The median time of clopidogrel treatment was 12 (4–30) months.

### Angiographic findings

Coronary angiography revealed three-vessel coronary disease in 15 (52%) patients. In 15 (52%) patients the culprit lesion for the qualifying acute MI was a de novo lesion not previously known or demonstrated. In 14 (48%) patients the culprit lesion was one previously treated by PCI and stent implantation, and in 7 (50%) of them stent thrombosis was the underlying pathological progression.

### Platelets function

Platelet function tests showed an average maximal ADP-induced platelet aggregation of 60 ± 17%. The average maximal arachidonic acid-induced platelet aggregation was 33± 24%. Nineteen (66%) patients had ADP-induced platelet aggregation more than 50% and were classified as clopidogrel poor responders. Twenty (69%) patients were aspirin non-responders. A dual antiplatelet poor response was seen in 16 (57%) patients. All 7 patients in whom the qualifying event was the result of a definite stent thrombosis had platelet non-response to chronic clopidogrel therapy.

### Matching results

Table 1 shows the characteristics, angiographic findings and platelet function of the 29 study patients and of the 58 matched clopidogrel-naïve patients. Despite matching for age, gender, prior cardiovascular history and type of index acute MI (STEMI versus NSTEMI), patients sustaining an acute MI while on chronic clopidogrel therapy were more likely to be diabetic (52% versus 33%, p = 0.1) and have more extensive angiographic coronary artery disease with a higher incidence of multi-vessel coronary artery disease (79% versus 55%, p = 0.03). Acute MI was more likely to reflect stent thrombosis among the clopidogrel-treated compared with the clopidogrel-naïve patients [7/29 (24%) versus 0/58 (0%), p<0.001]. Furthermore, clopidogrel-treated compared with clopidogrel-naïve acute MI patients had significantly higher ADP-induced platelet aggregation (69±12% versus 47±17%, p<0.001), and were more likely to show poor response (66% versus 38%, p = 0.02). Arachidonic acid-induced platelet aggregation among the clopidogrel-treated compared with the clopidogrel-naïve patients was not statistically different (32±18% versus 24±19% p = 0.09).

When patients were matched for age, gender, type of acute MI, history of cardiovascular disease and diabetes mellitus, clopidogrel-treated compared with clopidogrel-naïve acute MI patients still had significantly higher ADP-induced platelet aggregation (69±12% versus 43±13%, p<0.001), and were more likely to show poor response (66% versus 37%, p = 0.01). Arachidonic acid-induced platelet aggregation was not significantly different between the two groups (32±18% versus 24±14% p = 0.07).

## Discussion

In the present study we prospectively studied 29 consecutive patients who presented with acute MI despite chronic treatment with clopidogrel. Two-thirds of them presented with STEMI, and in one quarter, the infarction was related to definite stent thrombosis. Despite satisfactory compliance based on reports from the patients themselves, which were ascertained by HMO prescriptions, two-thirds demonstrated poor response to clopidogrel based on widely accepted definitions [17]. When the study patients were compared on a ratio of 1:2 with a group of clopidogrel-naïve acute MI patients matched for age, gender, cardiovascular history and type of acute MI (STEMI vs. NSTEMI), as a group the study patients demonstrated significantly higher clopidogrel platelet reactivity, and were significantly more likely to show poor response to clopidogrel.

We demonstrated that a high percentage of the ‘clopidogrel failure’ patients showed an inadequate response to clopidogrel. Similarly, incomplete inhibition of ADP-induced platelet aggregation has been demonstrated in a number of studies when patients were tested after the stent thrombosis occurred [18,19]. A previously published case series described 7 patients who presented with acute stent thrombosis while being treated with clopidogrel [20]. These patients exhibited a laboratory poor response to clopidogrel and 6 of them demonstrated the 2C19*2 genetic variant, associated with loss of function. Our report includes a larger number of patients with a wider spectrum of clinical presentations (i.e. other than acute stent thrombosis). In a case series by Pena et al [20], the median time of clopidogrel treatment was 6 days, a much shorter period than the patients in our series. Furthermore, the indications for clopidogrel treatment in our report included several other clinical scenarios other than coronary stenting. Accordingly, our current report further expands the significance of the association between the clinical phenomenon of ‘clopidogrel failure’ and clopidogrel poor responsiveness. On the other hand, high on-treatment platelet reactivity with clopidogrel was shown to be an independent predictor of the 1-year occurrence of stent thrombosis and recurrent cardiovascular events [21–23].

A recently published decade-long study of trends in acute MI characteristics demonstrated that the percentage of patients with STEMI decreased significantly from 1999 to 2009. In 1999, approximately two-thirds of all initial acute MIs were STEMI in nature, whereas by 2009, only two-fifths of all acute MIs were STEMIs [24]. Our study showed a much higher incidence of STEMI in patients presenting with ‘clopidogrel failure’. This finding might be explained, at least partially, by the relatively high prevalence of stent thrombosis in this group, which was much higher than reported with the 2^nd^ and 3^rd^ generation drug-eluting stents used in the current cohort [25,26].

Patients with diabetes have been shown to have a higher proportion of platelets expressing P-selectin and activated GP IIb/IIIa receptors than non-diabetic patients [27,28]. These patients may be less sensitive to inhibition by both aspirin and clopidogrel.

Regulatory agencies as well as major cardiac societies recommend the use of novel antiplatelet medications in patients with ACS [29,30]. Nevertheless, due to patent expiration in 2011, enormous generic competition, and the lack of data regarding the use of novel P2Y12 antagonists post PCI for stable coronary artery disease and cerebrovascular disease, clopidogrel is still the most commonly prescribed P2Y12 antagonist [14]. Thus our findings are still relevant even in the current era after the introduction of novel P2Y12 antagonists.

The present study has shown that, as a group, patients sustaining ACS despite prior clopidogrel therapy represent a group of patients who are likely to present with STEMI with a relatively high incidence of diabetes and extensive angiographic coronary artery disease. Furthermore, we found that patients sustaining ACS while on chronic clopidogrel therapy, compared to matched clopidogrel-naïve ACS patients, were much more likely to exhibit inadequate platelet inhibition to both clopidogrel and aspirin. The optimal approach to treat patients who develop ACS despite clopidogrel therapy is not well established. Screening for clopidogrel non-responsiveness and amending antiplatelet therapy accordingly has failed to improve clinical outcomes in a number of trials [31–33]. However, a previous study demonstrated that in patients with high on-treatment platelet responsiveness to clopidogrel, ticagrelor produced significantly higher platelet inhibition compared with prasugrel [34]. Furthermore, in the PLATO study which included 1397 patients on chronic clopidogrel treatment, the rate of events was reduced from 17.8% to 15.8% using ticagrelor over clopidogrel: p interaction equaled 0.43, meaning that ticagrelor’s superiority over clopidogrel was not affected by chronic treatment [35].

Limitations of this study include the fact that it was a single-center, observational study with a small cohort. Another potential limitation is the different times of platelet reactivity testing in the study groups. However, since some of the patients with clopidogrel hypo-responsiveness were reloaded with clopidogrel, the only time point that reflected platelet response to the chronic ingestion of clopidogrel was at admission. On the contrary, among clopidogrel-naïve patients, since some of the patients were treated with IIb/IIIa antagonists (Eptifibatide for 12–18 hours), 48 hours was the earliest time we could determine reactivity that reflected net platelet response to clopidogrel treatment. Variability in platelet response depending on the test used may exist and therefore evaluating platelet reactivity only by one technique in this study is a potential limitation of the study. Clopidogrel metabolite levels weren’t available during the study as a way to ascertain compliance and this may be an additional limitation of this study. Body mass index was not prospectively documented.

Our results suggest that platelet poor responsiveness to clopidogrel may contribute to the pathogenesis of the ACSs in ‘clopidogrel failure’. The small sample size and the lack of clopidogrel metabolite levels call for caution in generalization of our conclusion and therefore further research is warranted to examine this hypothesis.
